# Healthcare Experience of People with Acute Spinal Cord Injury: A Phenomenological Study

**DOI:** 10.3390/nursrep13040138

**Published:** 2023-12-04

**Authors:** Salomé Sobral Sousa, Maria João Andrade, Carla Sílvia Fernandes, Sara Rodrigues Barbeiro, Vanessa Taveira Teixeira, Rute Silva Pereira, Maria Manuela Martins

**Affiliations:** 1 Abel Salazar Institute of Biomedical Sciences, 4050-313 Porto, Portugal; salomesobral.neurocirurgia@chporto.min-saude.pt (S.S.S.); mariajoaoandrade.fisiatria@chporto.min-saude.pt (M.J.A.); up201802474@up.pt (R.S.P.); mmmartins@icbas.up.pt (M.M.M.); 2 Department of Neurosciences, University Hospital Center of Santo António, 4099-001 Porto, Portugal; sararodrigues.neurocirurgia@chporto.min-saude.pt (S.R.B.); vanessataveira.neurocirurgia@chporto.min-saude.pt (V.T.T.); 3 CINTESIS@RISE, Nursing School of Porto, 4200-072 Porto, Portugal

**Keywords:** spinal cord, hospitals, nursing, rehabilitation

## Abstract

Living with spinal cord injury (SCI) is a challenge that begins in the acute phase, when the disease, the limitations, and the treatments fill the days at the hospital. This study aims to understand the healthcare experience of the person with SCI in the acute phase, based on the Activities of Living Nursing Model (ALNM). It is a qualitative and phenomenological study based on the Standards for Reporting Qualitative Research. Data were collected via semi-structured interviews. Content analysis was performed using the ATLAS.ti software and Bardin’s methodology. The article was written following the COREQ guidelines. The categories were defined using the Roper–Logan–Tierney Model for Nursing. The sample included 16 people with incomplete SCI, different etiology, and neurological levels. Eleven of the twelve ALNM emerged from the interviews. The activities of mobilizing, eliminating, maintaining a safe environment, and communicating were emphasized the most. Controlling body temperature was not relevant. Mobility deficits and pain increased dependence. Feelings of motivation, encouragement, and frustration were highlighted. Professional expertise, rehabilitation resources, and support equipment promoted independence. The results in this sample revealed that people with SCI in the acute phase have complex challenges related to dependence awareness and treatments, but they always keep recovery expectations in mind.

## 1. Introduction

The challenge of living with a spinal cord injury (SCI) already begins in the acute phase. During this period, the injury, limitations, and treatments fill the days at the hospital. Acute management and rehabilitation of SCI depends on the level and type of injury, usually with initial treatment in an acute care setting, followed by prolonged treatment in a rehabilitation unit. The duration of the acute phase follows the natural history of neurorecovery, corresponding to 12 to 18 months after SCI [1]. People with SCI experience their new condition in an existential and complex context related to the abrupt and disrupted identity and break in their life projects [2]. The dependence condition brings feelings of loss and anticipates different changes, such as daily routines, lifestyle, and social roles, promoting a profound transformation at a personal level [3]. Denial, anger, and depression are stages that precede acceptance and adaptation to this new reality [4]. The suffering caused by the injury and the loss of autonomy may exponentiate the hospitalization experience, which exposes feelings of fear, sadness, insecurity, and depersonalization [5].

The hospital environment generates anxiety and anguish but is also seen as a space that relieves symptoms and promotes recovery [6]. In this context, the interactions between the person with SCI and the healthcare professionals, other patients, and family members will influence the expectations, the recovery experiences, and the quality of the transition from the hospital to the community [7].

From a nursing perspective, the patient with SCI can be understood by the Activities of Living Nursing Model (ALNM) because when a person is asked about what it means to live, regardless of their age or circumstances, they will mention activities such as eating and drinking, working and leisure, or sleeping. Twelve of the ALNM contribute to the complex process of life, influenced by a diversity of factors that will provide individuality. Throughout the life cycle, there is a dependency/independence continuum, acknowledging that there are stages in which it is not possible to perform certain activities independently, and this cannot interfere with the person’s dignity [8,9,10]. In various healthcare settings, all these concepts are valid—the model promotes integral care and greater knowledge about these moments and experienced realities, allowing nurses to intervene positively [9,10,11].

Considering the ALNM and the knowledge of the most affected areas in SCI patients [12], the following question emerges: how do SCI inpatients perceive the healthcare experience that supports their independence and autonomy in the acute phase? This pilot study aims to understand the healthcare experience of the person with SCI in the acute phase, based on the Activities of Living Nursing Model (ALNM), via their narratives that evoke descriptions, feelings, perceptions, expectations, motivations, or anxieties.

## 2. Materials and Methods

### 2.1. Design

An exploratory qualitative study was carried out using the phenomenological approach as a theoretical–methodological framework, which enhances the construction of the experiences lived by the participants [13]. Consolidated criteria for reporting qualitative research were used [14].

### 2.2. Participant Selection

This research was conducted in a Neurosurgery Department at a University Hospital in Portugal. The participants were selected according to an intentional convenience type sample. The inclusion criteria were adults diagnosed with SCI in the acute phase, admitted to a neurosurgery ward between 2021 and 2022, with a routine consultation at the institution scheduled from one to six months post-discharge, and participants who consented to the study and could communicate. The exclusion criteria were not consenting to the study, insufficient cognitive or oral communication skills, and not having post-hospital discharge consultation in the Neurosurgery Department.

### 2.3. Interviews

The data collection was carried out via semi-structured interviews with 16 participants. The interview script was subjected to a pilot test to validate the comprehensibility of the language and clarity of the questions. For this purpose, two previous interviews were carried out with people with SCI in the acute phase without being integrated into the study. The final interview script included questions for the participant’s sociodemographic characterization, such as gender, age, and profession. Additional information on each participant’s SCI characterization was taken from the electronic medical record. The remaining questions asked participants to describe their experiences and their evolution during hospitalization. The average duration of the interviews was 30 min. Priority was given to the face-to-face interview carried out by the main researcher and another research team member. All researchers are healthcare providers with experience in caring for people with SCI. Only when this contact was not possible was a video call made. The participants authorized the recording of the interview. Data saturation conditioned the total number of participants by verifying repetitive information to understand the phenomenon under study.

### 2.4. Data Analysis

The obtained information was fully transcribed and subsequently validated by each participant via email. Incorporating participant validation into the research process is important because it is a strategy to ensure the reliability of study data and results. It is also a way to address ethical concerns, be transparent, and place participants at the center of the research [15]. Qualitative content analysis was performed using ATLAS.ti 23.3.4 software (Thomas Muhr, Berlin, Germany) and Bardin’s methodology [16]. The interviews were coded, ensuring anonymity. The thematic-categorical content analysis was performed stepwise by the main researcher at three chronological points: pre-analysis, where via skim reading, it was possible to get a sense of the content; exploration of the material during which the content was organized, and initial ideas were systematized and codified; and lastly, inference and interpretation of results, where thematic grouping was carried out using the comparative method, according to pre-established categories based on the ALNM [9]. In this stage, three researchers met regularly to discuss categories and find consensus on codification. In the final stage, the results have been interpreted to answer the research question.

### 2.5. Ethical Considerations

The study presentation and the request for participation were carried out at the time of hospital admission. The study was subjected to the Ethics Committee of the institution’s assessment, with favorable assent nr. 2020.285 (217-DEFI/229-CE). All criteria of anonymity and confidentiality were followed.

## 3. Results

### 3.1. Participant’s Characterization

Table 1 shows the characteristics of the participants: nine males and seven females, aged between 26 and 78 years (Mean 52.9 SD 15.3). Eight participants have higher education. Five participants work in healthcare, and four are retired. Regarding family support, only 2 participants are living alone (P8; P9). Concerning the cause of hospitalization, the participants presented SCI of different etiologies, such as tumoral, degenerative, or traumatic. All injuries were incomplete; two cases were classified ASIA B, three ASIA C, and eleven ASIA D. The neurological level varied between C5 and S4, and lesions were found in the cervical, thoracic, lumbar, and sacral segments. Regarding the destination after acute hospitalization, nine interviewees received care in a specialized rehabilitation unit.

### 3.2. Experiences by People with Acute Spinal Cord Injury—Activities of Living

**Figure 1:**
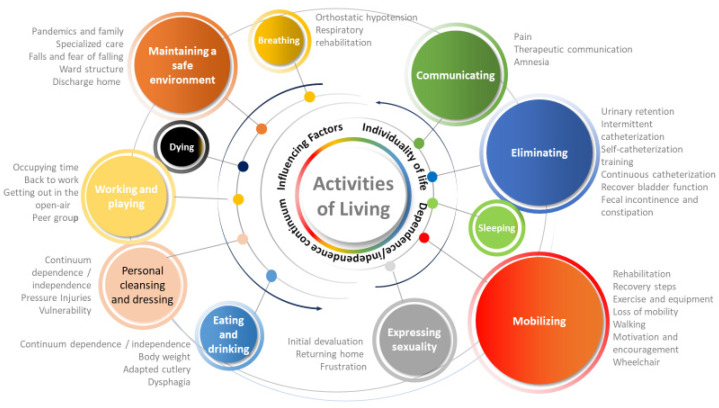
Experiences of the person with SCI in the acute phase.

Of the twelve ALNM identified by Roper, Logan, and Tierney, eleven emerged from the interviewee’s discourse and were identified as categories. The activities of mobilizing, eliminating, maintaining a safe environment, and communicating assumed greater relevance in the hospitalization experiences. Dying, sleeping, and breathing were highlighted less. The ALNM controlling temperature was not relevant to the participants’ experiences. The results can be seen in Figure 1.

#### 3.2.1. ALNM Mobilizing

This activity was central to the experiences and often influential in achieving the remaining ALNM. The following themes were isolated in this category:

- Rehabilitation (P1; P2; P3; P4; P5; P6; P7; P8; P9; P12; P13; P14; P15; and P16)—“I remember the first rehabilitation session with the nurse, the second, the third and remaining ones. I remember the contacts I had with the physiotherapist. All the methods and sessions were remarkable and important for my rehabilitation path” (P8).
- Stages of recovery (P2; P3; P4; P5; P6; P7; P8; P11; P12; P14; P15; and P16)—“I saw that my thumb was moving a little, and I thought—Stop! After all, it’s already moving. I held on to each evolution; each extra movement I could make was a victory” (P15).
- Exercises and equipment (P2; P3; P4; P5; P6; P7; P9; P12; P13; P14; and P15)—“I rode a kind of bicycle, and I have in my memory exercises on a balance board” (P5).
- Loss of mobility (P2; P3; P6; P8; P12; P13; P14; P15; and P16)—“After surgery, I immediately realized that my legs didn’t work” (P2).
- Walking (P1; P3; P4; P7; P11; P12; P14; and P15)—“The most exciting thing was to be able to take a few steps” (P11).
- Motivation and incentive (P5; P7; P8; P9; P14; P15; and P16)—“I am fully aware that I would not be able to recover if I didn’t also make an effort (…) it was not only the external strength that was needed but also the internal strength” (P14).
- Wheelchair (P3; P7; P11; and P13)—“When they brought me the wheelchair, I was in denial… I think I spent some days pretending that I was sitting in a normal chair” (P11).

#### 3.2.2. ALNM Eliminating

This activity is marked by the themes related to neurogenic lower urinary tract dysfunction and neurogenic bladder:

- Urinary retention (P2; P4; P5; P7; P8; P10; P11; P12; P13; P14; and P16)—“It was hard to accept that I couldn’t urinate on my own” (P2).
- Intermittent catheterization (P2; P5; P7; P8; P10; P11; P13; P14; and P16)—“regularly I had to remove the urine from the bladder, and then there was the water control… it was a kind of game where you had to drink, hour-by-hour” (P10).
- Self-catheterization training (P2; P4; P7; P8; P10; P11; P13; and P16)—“It wasn’t a big deal. The nurses helped to uncomplicate it” (P10).
- “Continuous catheterization” (P1; P5; P6; P15; and P16)—“Going home earlier but staying with the urinary catheter… that was horrible! How will I go out into the street? I like walking, hiking, doing my own things, being with my grandchildren” (P10).
- Recover bladder function (P1; P8; P12; and P15)—“While I was hospitalized, I did intermittent catheterization, but I always tried to urinate before. I started to be able to urinate and to have lower and lower bladder volumes, and I ended up recovering” (P7).
- Fecal incontinence and constipation (P1; P2; P3; P8; P11; P13; P15; and P16)—“, I had leaks during the effort at the gym, when going to the standing frame (…) I didn’t have an anal tampon” (P2).

#### 3.2.3. ALNM Maintaining a Safe Environment

This activity relates to safety issues, and the following themes emerge:

- Pandemic and family (P2; P3; P4; P5; P6; P11; P12; P13; P14; P15; and P16)—“without family nearby because the pandemic we could only have a visit every three days (…) it was such a difficult time I was going through” (P15), “having my mobile phone with me was essential to be able to talk to my wife” (P12).
- Specialization healthcare (P2; P4; P7; P8; P10; P12; P14; P15; and P16)—“I felt safe, in the hands of the professionals, who knew what they were doing” (P2), “the atmosphere of safety that was transmitted to me, makes me recovered” (P8).
- Falls and the fear of falling (P1; P3; P5; P7; P12; and P16)—“In the bathroom, I felt insecure, and was afraid of falling” (P12), “I would wake up and see the sides of the bed, and I felt closed, trapped” (P1).
- Ward structure (P7, P8, P13, and P14)—”The space does not have an adequate, safe structure designed for patients with mobility alterations and in rehabilitation” (P8).
- Discharge home (P5; P10; and P12)—“I preferred not to press for discharge because I felt safer in the hospital. The two days that I was overstaying were necessary to leave in a better condition. At home, I had no one to ask for help” (P12).

#### 3.2.4. ALNM Communicating

Communication is an integral part of human relationships and behavior. Pain is part of this ALNM, as it is identified via verbal and non-verbal communication. In this category, the following themes emerge:

- Pain (P1; P2; P3; P4; P5; P6; P8; P9; P10; P12; P14; P15; and P16)—“I felt an atrocious pain… but I woke up from surgery without any pain! Incredible. I couldn’t believe what was happening” (P10); “I remember at first I couldn’t move in bed because of the pain and thinking that so much pain was a sign that something was wrong, didn´t let me recover” (P14).
- Therapeutic communication (P2; P3; P8; P10; P11; P13; P14; and P16)—“I fell apart, I was devastated, I don’t even know what came to my mind! The rehabilitation nurse explained to me what was happening; she gave me strength, and from that moment on, I got things moving more quickly. I understood, I began to think that it had to be me who had to try and see this problem through to the end. Those words made me hold on more my problem” (P16).
- Amnesia periods (P1; P2; P3; P11; P13; and P14)—“I don’t remember anything from the hospital before the day I got up and took a few steps. That was the key moment for me” (P11).

#### 3.2.5. ALNM Working and Playing

The ALNM Working and Playing, even in an acute context, can be observed in the daily routine at the hospital. Four thematic areas emerged in this category:

- Occupying time (P1; P2; P5; P6; P7; P8; P9; P10; P12; P13; P14; and P15)—“I made an agenda and organized myself, I’d wake up, go to the internet while waiting for the bath and breakfast. Then, I’d be reading or talking on the phone (…), and then it was time to do gymnastics. Then came lunch, and then I had to rest (…) I’d get well and go training again all afternoon. The day passed quietly and quickly” (P9), “I think it is missing the rehabilitation at the weekend because we spend that time there, very still” (P2).
- Back to work (P2; P6; P8; P9; P12; and P14)—“I expect to work in the future, but certainly I won’t be able to continue doing the same thing I used to do” (P6).
- Getting out in the open air (P5; P7; P11; and P14)—“The first day they brought me to the terrace, it was awesome. I even got emotional (…) one of the best things was feeling the sun” (P11).
- Peer group (P2; P3; and P7)—“I felt support in the hospital talking to other patients who share the same problems” (P2).

#### 3.2.6. ALNM Personal Cleansing and Dressing

In addition to personal cleansing and dressing, this category includes skin conditions and prevention of pressure injuries:

- Dependence/independence continuum (P2; P3; P6; P7; P11; P13; P14; P15; and P16)—“The thing that gave me the most joy was when I could go to the bathroom by myself to shave, wash, and take a bath” (P16).
- Pressure injuries (P2; P3; P7; P8; P13; P14; and P16)—“I got a change in the chest area, which was very red, very hard. It would have been the position and time of the surgery” (P14), “I’m on a proper mattress and with a cushion on the chair” (P13).
- Vulnerability (P3and P11)—“Once, in the rehabilitation center, I was coming from the gym, very sweaty, and I asked if I could take a quick shower, and they told me that as I was in a wheelchair, I couldn´t take a bath whenever I want it” (P3).

#### 3.2.7. ALNM Expressing Sexuality

The ALNM Expressing Sexuality is developed along four thematic lines:

- Initial devaluation (P2; P4; P9; P11; P14; P15; and P16)—“During hospitalization, I never thought about sexuality. My focus was to get well and my husband’s too” (P11).
- Return home (P2; P6; P12; P15; and P16)—“The first weekend I went home, my husband was even afraid to touch me. Then when I went home again, we still tried it, but I had no sensibility” (P2).
- Frustration (P15)—”It is a difficult and slow process… we are insisting, insisting and then it is a frustration. The human being is very complex!” (P15).

#### 3.2.8. ALNM Eating and Drinking

This activity plays a significant role in daily life patterns. In this category, narratives emerge about the following:

- Dependence/independence continuum (P3; P8; P11; P14; and P15)—“As I had altered motricity, I needed help to cut food; I remember wanting to eat an apple and having to ask someone to cut it. I was recovering and when I discharge, I already eating alone” (P14).
- Body weight (P3; P7; and P10)—“I lost about 3 or 4 Kg when I had the surgery, and I realized that being less heavy makes it easier to help me” (P10).
- Adapted cutlery (P11 and P15)—“When they brought me cutlery with adaptors, then I really felt in denial… I needed time to adapt myself to the idea” (P11).
- Dysphagia (P3 and P14)—I had difficulty swallowing, and having the cervical collar made it even worse” (P14).

#### 3.2.9. ALNM Breathing

The activity includes issues related to the cardiorespiratory system, such as

- Orthostatic hypotension (P2; P6; P11; P13; and P14)—“I had very low blood pressure, and it got worse when I got up; it was a tough phase at the beginning” (P2).
- Respiratory rehabilitation (P2; P8; P13; P14; and P16)—“I did those exercises to control breathing and used the spirometer. I still do today. I wasn’t breathing in the best way…” (P16).

#### 3.2.10. ALNM Sleeping

This ALNM (P3; P9; and P12) was not always possible to satisfy due to the noise—“the agitated patient didn’t let anyone sleep!” (P9).

#### 3.2.11. ALNM Dying

The ALNM Dying was only mentioned by one participant (P5), having been precipitated by the death of a patient in the room: “There was one thing that disturbed me a lot; I even cried that night. It was a patient who was in front of me, a young man who died. That night, I felt I was occupying a space that was not mine (…) He still had so much to live, and I have already lived so much, and I am still here. But life and death are like that” (P5).

## 4. Discussion

Via a phenomenological approach, this study aimed to understand the process of experiencing ALNM during the acute phase in people with SCI. A set of categories emerged from the results, corresponding to eleven of the activities of living in Roper, Logan, and Tierney’s Model.

The ALNM Maintaining a Safe Environment concerns identifying hazards in the surrounding environment (including healthcare settings) and how they may threaten individual safety and well-being [10]. As an inpatient, the specialization of care in dedicated SCI units and reaching better outcomes has been associated with feelings of safety and increased trust [17]. Post-SCI, the recovery phase relies heavily on family support [18,19]. Fear and anxiety among inpatients in this phase may be exponentiated when, for safety reasons, the environment calls for less in-person contact and increased social distancing, such as that experienced during the pandemic of COVID-19 [20]. As in this study, the family’s absence from the hospital brought feelings of isolation, and although virtual contact does not replace the real world, using technology to maintain contact with relatives was considered necessary [21]. In a population with an increased risk of falls [22], falls and fear of falls are also insecurity generators. It is essential to promote adherence to multifactorial programs to reduce the incidence of falls and, above all, to decrease the severe associated consequences [23]. For safety, it is also essential to provide an accessible environment. In this study, participants also provided comments and opinions along these lines. The complex condition of people with SCI, such as dependence on a wheelchair, requires a well-prepared structure without physical barriers, which can be found in rehabilitation units [24,25]. When available, assistive technology, such as robotic devices, improves independence as it allows the person with SCI to have greater control over the environment and use everyday objects easily and safely [25]. In the final phase of hospitalization, expressions of insecurity related to hospital discharge were found in this study. On the one hand, returning home is one of the main goals for the inpatient. On the other hand, the feelings of security associated with professional care led to the impression of less follow-up in shorter hospitalizations, generally associated with SCI of a non-traumatic cause [26]. Timely preparation for discharge, family support, and access to specialized rehabilitation programs were associated with less stress during the home transition [12].

The ALNM Communicating is an integral part of human relationships and behavior, and any information regarding activities of living is shared via communication [9]. The health professional is a communication activator agent and plays a fundamental role in the interactions between the patient and the family [27]. In this study, communication with health professionals was valued by the patients. In the initial post-SCI period, health professionals must facilitate clearer and continuous conversations considering barriers like stress, denial, and difficulty in retaining information by the patient [28,29,30]. Periods of amnesia are also reported in this study. In addition, although patients receive some information, it may be difficult for them to understand its real implications [29]. This population must be treated by experienced professionals with appropriate sensitivity and honesty, able to give hope while remaining realistic about the possibility of recovery [28,29,30,31]. Within the ALNM Communicating, pain has been shown to be one of the most severe and disabling complaints after SCI [12,32,33]. In this study, there were several experiences of pain. In the initial week of rehabilitation, the intensification of physical activity is believed to impact pain increment. Mismanaged pain can reduce the effectiveness of the treatments, extend the length of stay, and decrease satisfaction with the provided care [34]. Various pharmacological and non-pharmacological treatments can reduce not only pain but also anxiety, depression, and fatigue and improve muscle strength and range of motion [32,33,35].

The ALNM breathing was not very valued in the experiences of the participants. It is known, however, that orthostatic hypotension occurs in around 70% of people with cervical or high dorsal SCI and can cause intolerance to mobilization and lifting, representing a significant obstacle in the progression of the rehabilitation program [36]. Compressive stocks, early mobilization, and progressive standing orthostats on a tilting table are some strategies that can be used in the acute phase of SCI.

When affected, the ALNM Eating and Drinking can make a person dependent on others to support and assist with meeting nutritional and hydration needs [10]. Situations of dependence were experienced in this study. The development of adaptive strategies is essential at this stage. However, signs of rejection, such as denial, can be identified [37]. In this context, peer groups can have a very positive influence via their real-life example, helping to unblock triggered negative feelings and encouraging self-care skills [37,38]. Eating and drinking habits can affect other activities of living—overeating results in obesity and can decrease the capacity to mobilize [10]. Body weight, as a physical dimension reflecting this ALNM, can indicate the nutritional needs of the person with SCI, which are complex and influenced by factors such as injury stage and activity level [39]. Weight gain and a sedentary lifestyle increase cardiovascular risk, which is already exceptionally high in this population [40].

The ALNM Personal Cleansing and Dressing is affected when motor skills, such as balance, coordination, range of motion, muscle strength, and even manual dexterity, are compromised after SCI [41]. The interviewees experienced dependence on this ALNM. These skills should be learned during the initial rehabilitation period and reach their maximum level of independence before hospital discharge [37]. Self-care behaviors, incorporated into the daily routine, promote active participation and are essential to prevent complications and improve health conditions and well-being [37,42]. Pressure ulcers associated with this ALNM are the second most frequent secondary complication in people with SCI during the acute phase. Besides affecting the quality of life, they increase length-of-stay, morbidity, and mortality [43,44]. The most important strategy for prevention is to improve skin self-care, positioning, transfer techniques, and weight control skills [44].

The ALNM Mobilizing was a protagonist in the experiences reported in this study. The reduction or loss of sensorimotor and autonomic functions caused by SCI results in low levels of physical activity, often aggravated by the need for bed rest, either due to hemodynamic, spinal cord, or surgery-related instability [37]. It can cause devastating effects on physical and psychological well-being, with a substantial impact on quality of life [10]. Physical activity is crucial after SCI to increase independence and is described as a way of maintaining well-being and coping with the new condition [37], especially among people with limited recovery [7]. Feeling in control of movement can be a source of autonomy. In this study, the ability to walk was a central experience, a source of joy and concern. The first contact with the wheelchair is a milestone in the awareness of a new condition, which involves a symbolic confrontation with a new identity [24]. This moment was also a milestone for the participants in this study. People with SCI learn to move their bodies in a new way [37], often requiring adaptive strategies, support equipment, and specific learning approaches. Healthcare professionals strongly recognize that improving motivation and self-confidence is essential and required to generate further progress [30,37]. They should emphasize participation in rehabilitation via focus and attention on what the person is actually able to do [28].

The ALNM Working and Playing can be limited or worsened by reasons such as disability, illness, injury, or the aging process [10]. Hospitalized patients with SCI may feel bored and depressed during periods when they feel there is nothing to do in the hospital [29]. The possibility of increasing rehabilitation time, performing exercises during the night and on weekends, has already been mentioned in previous studies as essential, not only to enhance the rehabilitation process but also as a need to fill time during hospitalization [21]. This was also a need felt in this study. Returning to work, albeit with adaptations, was a concern for the participants. It is well established that one of the best indicators of rehabilitation effectiveness after SCI is successfully returning to work, and this area requires a more significant emphasis during the rehabilitation process [45].

The ALNM and Expressing Sexuality relates to issues that are beyond sex and sexual relationships, is an essential component of adult relationships, and is also expressed in personality and behavior [10]. As found in this study, sexual health can sometimes be overlooked during the acute phase as patients, families, and healthcare professionals focus efforts on clinical stabilization and regaining independence in self-care and mobility [46,47,48]. This was also a reality in this study. Although participants’ sexual disorders are predominantly physical, the psychological dimension is also influenced [47]. In a previous study, people who did not consult healthcare professionals for sexual problems expressed lower expectations and levels of hope for the future [46].

Sleeping is vital and an essential ALNM [9]. In line with the results of this study, evidence demonstrates that people experience disturbed sleep during hospitalization [49]. Intervening in modifiable factors, such as reducing light and noise, may help reduce fatigue and improve sleep experiences in the hospital.

Dying is the last act of living. Dying suddenly from a natural cause in old age is generally considered a ‘good death’ [8]. The ALNM Death and Dying was only mentioned by one participant associated with the death of a young person during hospitalization at an early stage in his life plan.

The study presented limitations related to the unique context where the study was carried out and the large amount of data obtained, which made it complex to explore all participants’ experiences.

## 5. Conclusions

This study allowed us to understand the process of experiencing ALNM in hospital settings in people with SCI during the acute phase. This study will help rehabilitation nurses provide care to future inpatients. Participants expressed the importance of access to specialized care and the possibility of participating in rehabilitation programs tailored to their acute condition. It is possible to understand that people with SCI face several challenges during the hospitalization process associated with loss of autonomy and independence, the need to overcome and adapt, and the hope for a complete recovery.

The ALNM Mobilizing, Eliminating, Maintaining a Safe Environment, and Communicating were the most emphasized ALNM regarding hospitalization experiences. Dying, Sleeping, and Breathing were less highlighted, and the activity of Controlling Body Temperature was not mentioned. A close association was observed among ALNM, mainly for “mobilizing”, which affected all the other categories. Mobility deficits associated with SCI are the cause of the change of condition and, consequently, the basis of all dependence. Based on their experiences, participants expressed their opinions on safety issues that gave rise to specific aspects of insecurity, and they also valued the nurses’ role in care. The communication established by the team and the participation-friendly environment were highlighted as essential. The time spent in the hospital created space to value work and leisure. Issues around sexuality at a very early stage were not valued. Pain was a factor that interfered with activities of living. Motivation, encouragement, and frustration were feelings emphasized during rehabilitation. Specializing professionals, rehabilitation resources, and support equipment promoted independence and autonomy.

Hospitalization is described as a continuum of dependence and independence in each ALNM, with a synchronized effort with the healthcare team to aim for a safe return home. The individuality of life was perceived in the unique experiences each participant faced with hospitalization and SCI, as well as in the personalized responses to their needs. In the rehabilitation context, it is crucial to consider the implications of these patient experiences for developing personalized care plans. Further research on this topic is needed to develop strategies and policies that promote the autonomy and independence of SCI patients during hospitalization.

## Figures and Tables

**Table 1:** Participant’s characterization.

Demographic and Injury Characteristics of Participants								
SCI Patients	Gender	Age	Occupation	Educational Level	Diagnosis	ASIA Classification	Neurological Level	Post-Discharge
**P1**	Female	73	Retired	1st cycle of basic education	Schwannoma	D	T12	Physiatry department
**P2**	Female	52	Educator	Higher education	Pott’s disease	B	T9	Physiatry department
**P3**	Female	52	Nurse	Higher education	SCIWORA	D	C5	Physiatry department
**P4**	Female	26	Alternative medicine therapist	Secondary education	Discal hernia	D	S1	Home
**P5**	Male	78	Retired	Higher education	Cauda equina syndrome	D	L5	Home
**P6**	Male	40	Warehouse operator	3rd cycle of basic education	Epidural neoplasm	C	C5	Physiatry department
**P7**	Male	57	Retired	3rd cycle of basic education	Conus medullaris syndrome	C	L2	Physiatry department
**P8**	Female	47	Nurse	Higher education	Schwannoma	C	T2	Physiatry department
**P9**	Female	52	Physiotherapist	Higher education	Schwannoma	D	L2	Home
**P10**	Female	66	Administrative	2nd cycle of basic education	Herniated disc	D	S2	Home
**P11**	Female	57	Unemployed	1st cycle of basic education	Cervical contusion	D	C5	Home
**P12**	Male	33	Researcher	Higher education	Vertebral hemangioma	D	L2	Home
**P13**	Male	73	Retired	1st cycle of basic education	Epidural neoplasm	B	T4	Home
**P14**	Female	26	Nurse	Higher education	Schwannoma	D	C5	Physiatry department
**P15**	Male	54	University Higher Technician	Higher education	Cervical Spondylotic Myelopathy	D	C5	Home
**P16**	Male	60	Driver	2nd cycle of basic education	Ependymoma	D	T3	Physiatry department

## Data Availability

Data from this study are available upon request from the corresponding author.

## Public Involvement Statement

No public involvement in any aspect of this research.

## Guidelines and Standards Statement

Consolidated criteria for reporting qualitative research (COREQ) checklist.
